# Identification of natural antimicrobial agents to treat dengue infection: *In vitro* analysis of latarcin peptide activity against dengue virus

**DOI:** 10.1186/1471-2180-14-140

**Published:** 2014-05-31

**Authors:** Hussin A Rothan, Hirbod Bahrani, Noorsaadah Abd Rahman, Rohana Yusof

**Affiliations:** 1 Department of Molecular Medicine, Faculty of Medicine, University of Malaya, 50603 Kuala Lumpur, Malaysia; 2 Department of Pharmacology, Faculty of Medicine, University of Malaya, 50603 Kuala Lumpur, Malaysia

**Keywords:** Latarcin peptide, Dengue virus, NS2B-NS3 Protease, Protease activity, Viral inhibition

## Abstract

**Background:**

Although there have been considerable advances in the study of dengue virus, no vaccines or anti-dengue drugs are currently available for humans. Therefore, new approaches are necessary for the development of potent anti-dengue drugs. Natural antimicrobial peptides (AMPs) with potent antiviral activities are potential hits-to-leads for antiviral drug discovery. We performed this study to identify and characterise the inhibitory potential of the latarcin peptide (Ltc 1, SMWSGMWRRKLKKLRNALKKKLKGE) against dengue virus replication in infected cells.

**Results:**

The Ltc 1 peptide showed a significantly inhibitory effect against the dengue protease NS2B-NS3pro at 37°C, a physiological human temperature, (IC_50_, 12.68 ± 3.2 μM), and greater inhibitory effect was observed at 40°C, a temperature similar to a high fever (IC_50_, 6.58 ± 4.1 μM). A greater reduction in viral load (p.f.u./ml) was observed at simultaneous (0.7 ± 0.3 *vs.* 7.2 ± 0.5 control) and post-treatment (1.8 ± 0.7 *vs.* 6.8 ± 0.6 control) compared to the pre-treatment (4.5 ± 0.6 vs. 6.9 ± 0.5 control). Treatment with the Ltc 1 peptide reduced the viral RNA in a dose-dependent manner with EC_50_ values of 8.3 ± 1.2, 7.6 ± 2.7 and 6.8 ± 2.5 μM at 24, 48 and 72 h, respectively.

**Conclusions:**

The Ltc 1 peptide exhibited significant inhibitory effects against dengue NS2B-NS3pro and virus replication in the infected cells. Therefore, further investigation is necessary to develop the Ltc 1 peptide as a new anti-dengue therapeutic.

## Background

Dengue is a viral disease caused by four serotypes of the *Flavivirus* genus [1] and is prevalent in tropical and subtropical countries, ranging from Southeast Asia to the Americas [2]. Over 390 million people are infected with dengue virus (DENV) annually in over 100 counties, resulting in approximately 12000 deaths [3]. In Malaysia, the fatality rate of dengue infection is approximately 3.6% based on the total number of dengue infections. The majority of deaths caused by dengue infection occur after the mild infection develops into severe haemorrhagic fever and dengue shock syndrome [4]. In addition to the global health problem caused by dengue infection, it also has an economic burden. The estimated cost of dengue infection is approximately US$ 950 million per year, which is higher than hepatitis B and Japanese encephalitis in Southeast Asia [5].

DENV is an enveloped virus with a positive stranded RNA genome of approximately 11 kb in length that encodes a single polypeptide. The host cell furin and the viral NS2B-NS3 serine protease NS2B-NS3pro cleave the viral polyprotein at different positions to release viral structural and non-structural proteins [6-9]. Therefore, the viral NS2B-NS3pro is a potential target for the design and development of antiviral drugs [10,11]. NS2B acts as necessary a co-factor for the optimal catalytic activity of NS3 [10,12]. NS2B-NS3pro consists of 185 residues from the N-terminal of the NS3 protein and the central 44 residues of the hydrophilic domain of NS2B [12,13].

Despite the numerous studies about dengue virus, currently, no effective vaccine or antiviral therapeutics is available [14,15]. It is difficult to develop anti-dengue treatments because of the incidence of the antibody-dependent enhancement due to the existence of four dengue serotypes, the unavailability of an actual animal model [16,17] and the nature of the dengue protease, a promising target for dengue inhibitor development, which possesses a flat and hydrophilic active site that decreases the possibility of finding potent inhibitors to develop as antiviral therapeutics [18]. These facts accentuate the need for new approaches to develop potent anti-dengue drugs.

Natural antimicrobial peptides (AMPs) are produced in the majority of living organisms as protection against various pathogens, including viruses. We hypothesise that AMPs that possess potent antiviral activities may be considered as hits-to-leads for developing new antiviral drugs. Therefore, the objective of this study was to identify and characterise the inhibitory potential of the latarcin peptide (Ltc 1, SMWSGMWRRKLKKLRNALKKKLKGE) against dengue virus replication in human cells. Ltc 1 is one of approximately seven latarcin peptides, which are produced in the venom gland of *Lachesana tarabaeve*, a central Asian spider. Recent studies showed considerable antimicrobial activities of the latarcin peptides against bacteria and yeast [19-21]. In particular, the Ltc 1 peptide showed moderate haemolytic activity and significant antimicrobial activity compared to the other latarcin analogues [20]. However, there is a paucity of available data on the antiviral activities of Ltc 1 peptide. This study demonstrates for the first time significant inhibition by Ltc 1 against dengue NS2B-NS3pro and dengue virus replication in HepG2 cells.

## Methods

### Virus propagation in mosquito cells and titration

HepG2 cells with passage number less than 60 were maintained in DMEM medium supplemented with 10% FBS and incubated at 37°C in 5% CO_2_. HepG2 cells were used to study the peptide cytotoxicity and antiviral activity. Dengue virus serotype-2 (DENV2) was first propagated in C6/36 cells. The DENV2-infected cells that showed cytopathic effects (CPE) were lysed with a freeze and thaw cycle. The culture medium was then centrifuged at 1800 rpm for 10 min to remove the cell debris, filtered (0.2 μm), portioned into aliquots and stored at -80°C until use. The viral titre of the DENV2 suspension was established by serial dilutions on Vero cells using a plaque assay.

### Peptide synthesis

The Ltc 1 peptides were manufactured chemically using standard solid-phase peptide synthesis with a Symphony parallel synthesiser (Protein Technologies, Tucson, AZ, USA) as previously described [22]. Briefly, the aqueous phase of the peptide synthesis was lyophilised to yield the crude peptide. The identity of the crude peptide was confirmed by LC-MS, and purification of the crude peptide was performed by RP-HPLC (Agilent 1200 series, USA). The identity of the 98% pure purified peptide was confirmed by LC-MS (Shimadzu LC/MS 2020, single quad, Japan). The purified peptide was then lyophilised using a Savant AES 2000 Automatic Environmental SpeedVac system. To prepare 2 mM pure peptide, 6.14 mg lyophilised peptide was dissolve into 1 ml filtered-deionised water for use as a stock solution.

### Protein-protein docking

The interaction between the Ltc 1 peptide and dengue NS2B-NS3pro was identified by protein-protein docking study. The Protein Data Bank (PDB) files of Ltc 1 (2PCO) and NS2BNS3pro (4M9F) were used in rigid global docking using an available online server (FireDoc, http://bioinfo3d.cs.tau.ac.il/FireDock/refs.html) as described previously [23,24]. The results of the protein-protein docking were further analysed using Discovery Studio software version 3.5.

### ELISA binding of Ltc 1 to dengue NS2B-NS3pro

Enzyme-linked immunosorbent assay (ELISA) was used to examine the binding affinity of Ltc 1 to dengue NS2B-NS3pro. Increasing concentrations of purified dengue NS2B-NS3pro (0, 20, 30 and 50 nM/well) in carbonate/bicarbonate buffer (Sigma, USA) were bound to black 96-well plate with transparent bottom at 4°C overnight in triplicates. The wells were blocked with PBS containing 0.05% Tween 20 (PBS-T) plus 0.5% BSA for 1 h at room temperature and washed three times with PBS-T. Increasing concentrations of the Ltc 1 peptide labeled with FITC fluorescence dye (0, 0.1, 0.5, 1, 5, 10, 20, 30, 50 nM) were prepared in PBS-T plus BSA; 100 μl of each dilution of the Ltc 1 bound to plates for 3 h on ice in dark place. After the plates were washed, the fluorescence signals of bound Ltc 1 were detected using Tecan Infinite M200 Pro fluorescence spectrophotometer (Tecan Group Ltd., Switzerland).

### Dengue NS2B-NS3 protease (NS2B-NS3pro) assay

The NS2B-NS3pro assay was performed to examine whether the Ltc 1 peptide inhibits the DENV2 serine protease [12,25]. Briefly, a single chain NS2B (G_4_-T-G_4_) NS3pro was produced as a recombinant protein in *E. coli* as previously described [22]. The end point reaction mixture was performed in black 96-well plates, which contained 2 μM recombinant NS2B-NS3pro, 100 μM fluorogenic peptide substrate (Boc-Gly-Arg-Arg-AMC) and varying concentrations of the Ltc 1 peptide (0.1 to 40 μM) buffered at pH 8.5 with 200 mM Tris-HCl in a total volume of 200 μl. The reaction mixtures without peptide, substrate with the peptides, enzyme and different concentrations of the peptides were used as controls. Thereafter, all reaction mixtures were incubated at either 37°C or 40°C for 30 min, and the substrate was added to the specific reaction mixtures and incubated at the same temperatures for an additional 30 min. Measurements were performed in triplicate using a Tecan Infinite M200 Pro fluorescence spectrophotometer (Tecan Group Ltd., Switzerland). Substrate cleavage was normalised against the buffer only (control) at an emission wavelength of 440 nm with excitation at 350 nm. The fluorescence values obtained with the no-inhibitor control (0.0 μM peptide) were set at 100%, and those in the presence of peptide were calculated as a percentage of the control using non-linear regression in GraphPad Prism (version 5.01) software. The IC_50_ was calculated from nonlinear regression fitting of the signal *vs.* concentration data points to the standard dose–response equation *Y* = *Bottom* + (*Top* - *Bottom*)/(1 + 10^^^((*X* - *LogIC*50)))*.* In this equation, *X* is the log of the compound concentration, *Y* is the response signal, and the bottom and top refer to the plateaus of the sigmoid response curve. All assays were performed in triplicate and repeated twice. The inhibition percentage was calculated using the following formula:

$$\begin{array}{l} \%\;\mathrm{DENV}2\;\text{protease activity}=\\ 100-\left(\frac{\left(\text{intensity of enzyme activity}–\text{intensity left after inhibition}\right)}{\text{intensity of enzyme activity}}\right)\;\times 100 \end{array}$$

### Ltc 1 peptide cytotoxicity

The cytotoxicity of the Ltc 1 peptides was evaluated by determining the maximal non-toxic dose (MNTD) and the 50% cytotoxic concentration (CC_50_) of the cells using the Non-Radioactive Cell Proliferation assay (Promega, USA) according to the manufacturer’s instructions. The peptide concentration of 25 μM showed 80% cell viability and was considered the MNTD value, assuming that approximately 80% of the cells were healthy. Vero cells were seeded at 1×10^4^ cells/well in triplicate under optimal conditions (37°C, 5% CO_2_ in a humidified incubator) in 96-well plates with blank controls (media only) and cell controls (cells only). After an overnight incubation, the cells were treated with increasing concentrations of Ltc 1 peptide (0, 4, 8, 16, 32, 64 and 120 μM) with DMEM medium supplemented with 2% FBS and the cell culture was analysed after 72 h. The percentage of cell viability was calculated as follows: 100 - (absorbance of treated cells/absorbance of untreated cells) × 100. The MNTD and CC_50_ values were calculated from the dose-response curves.

### Real Time Cell Proliferation Assay (RTCA assay)

This assay was performed to test the real time effects of the Ltc 1 peptide on cell viability. Cell proliferation was measured using the xCELLigence Real-Time Cellular Analysis (RTCA) system (Roche, Germany) as described previously [26]. Cell viability and growth were monitored continuously after applying increasing concentrations of the Ltc 1 peptide (0, 12.5, 25, 50, 100, 150, 200, 250 μM). Briefly, the background measurements were recorded after adding 100 μl culture medium to the wells. Next, the cells were seeded at a density of 1 × 10^4^ cell/well in a 16-well plate with electrodes for 18 h to allow the cells to grow to log phase. The cells were treated with different concentrations of peptide dissolved in cell culture medium and continuously monitored for up to 100 h. The cell sensor impedance was expressed as an arbitrary unit named the cell index. The cell index was recorded every 5 minutes using a RTCA analyser. To eliminate variation between the wells, the cell index values were normalised to the value at the beginning of the treatment.

### Treatment of DENV-infected cells with the Ltc 1 peptide

To infect the HepG2 cells with DENV2, the cells were cultured in 24-well plates (1.5 × 10^5^ cells/well) for 24 h at 37°C and 5% CO_2_. The virus supernatant was added to the cells at a MOI of 2, followed by incubation for 1 h with gentle shaking every 15 min for optimal virus to cell contact. The cells were washed twice with fresh serum-free DMEM after removal of the virus supernatant. Then, fresh complete DMEM containing 25 μM Ltc 1 peptide was added to the cultures and incubated for 72 h. The HepG2 cells were then collected, and the virus particles and expression level of the viral NS1 protein were examined using immunostaining and western immunoblotting.

### Time-of-addition assay

This assay was performed to identify the mode of antiviral activity of the Ltc 1 peptide against DENV2 entry, replication and release from the infected cells. Three independent experiments were performed in triplicate for pre-, simultaneous and post-infection treatments. HepG2 cells were grown in a 24-well tissue culture plate (1.5 × 10^5^ cells/well), incubated 24 h under optimal conditions and infected with DENV2 at an MOI of 2. For pre-treatment infection, 25 μM peptide was added to the cells before virus inoculation and incubated for 24 h. After removal of the old medium containing the peptide, the DENV2 supernatant was added, followed by incubation for 1 h with gentle shaking every 10 min for optimal virus to cell contact. The virus supernatant was removed and the cells were washed twice with fresh serum-free DMEM medium to remove the residual virus. Fresh complete DMEM medium was added and the cultures were incubated for 72 h at 37°C, supplemented with 5% CO_2_. Identical applications were performed for the simultaneous treatment, except the peptide was mixed with the virus supernatant and incubated at 37°C for 1 h, and then inoculated onto the HepG2 cells. The post-treatment infection was performed after inoculation of the HepG2 cells with DENV2, and complete DMEM medium with the Ltc 1 peptide was then added. The cultures including the peptide were incubated for 72 h at 37°C and 5% CO_2_, and three wells of infected cells in each experiment were maintained without treatment as controls. The cell supernatants were collected and stored at -80°C for viral load determination using a plaque formation assay.

### Dose-response assay

This assay was performed to evaluate the 50% effective concentration (EC_50_) of the Ltc 1 peptide against DENV2. HepG2 cells were grown in six-well microplates (1.5 × 10^6^ cells/well) for 24 h in quadruplicate experiments. The cell culture media were removed and the cells were washed three times with PBS. Then, fresh medium containing the virus supernatant was added at MOI of 2, followed by incubation for 1 h with gentle shaking every 15 min. The viral residues were removed by washing with PBS, and serial dilutions of the Ltc 1 peptide (0, 2.5, 5, 10, 20, 40, 80 μM) were added. The cultures including the peptide were incubated for 72 h at 37°C and 5% CO_2_. The cell supernatants were collected and stored at -80°C for viral load determination using viral RNA and were quantified using one step qReal time-PCR.

### Virus quantification by plaque formation assay

To determine the virus yield after treatment with different concentrations of peptide, the culture supernatants were collected and serially diluted to reduce the effects of the drug residues. A 10-fold serial dilution of medium supernatant was added to new Vero cells grown in 24-well plates (1.5 × 10^5^ cells) and incubated for 1 hr at 37°C. The cells were then overlaid with DMEM medium containing 1.1% methylcellulose. The viral plaques were stained with crystal violet dye after a five-day incubation. The virus titres were calculated according to the following formula:

$$\begin{array}{ll} \mathrm{Titre}\;\left(\mathrm{pfu}/\mathrm{ml}\right)= & \text{number of plaques}\times \text{volume of}\\ & \text{diluted virus added to the well}\times \\ & \text{dilution factor of the virus used}\\ & \text{infect to the well in which the}\\ & \text{plaques were enumerated}. \end{array}$$

### Western blot

Cells lysates were prepared for immunoblotting against dengue viral antigen using ice-cold lysis buffer. The amount of protein in the cell lysates was quantified to ensure equal loading (20 μg) of the western blot gels using the 2-D Quant Kit (GE Healthcare Bio-Sciences, USA) according to the manufacturer’s instructions. The separated proteins were transferred onto nitrocellulose membranes and then blocked with blocking buffer. The membrane was incubated overnight with anti-DENV2 antibody specific to the viral NS1 protein (Abcam, UK, Cat. no. ab41616) and an anti-beta actin antibody (Abcam, UK, Cat. no. ab8226). After washing three times, the membranes were incubated with anti-mouse IgG conjugated to horseradish peroxidase (Dako, Denmark) at 1:1,000 for two h. Horseradish peroxidase substrate was added to for colour development.

### Indirect immunostaining

To examine the efficacy of the Ltc 1 peptide for reducing viral particles, HepG2 cells were grown on cover slips in 6-well plates and infected with DENV2 at an MOI of 2. The DENV2-infected cells were then treated with 25 μM peptide for 24 h. The cells were washed three times with PBS to remove the peptide residues and then fixed with ice-cold methanol for 15 min at -20°C. After washing, the cells were incubated with coating buffer for 1 h at room temperature. A mouse antibody specific to the dengue envelop glycoprotein (Abcam, UK, Cat. no. ab41349) was added, and the cells were incubated overnight at 4°C. The cells were washed three times with PBS and incubated for 30 min with an anti-mouse IgG labelled with FITC fluorescent dye (Invitrogen, USA, Cat. no. 62-6511). To stain the cell nuclei, Hoechst dye was added (Invitrogen, USA, Cat. no. H1399) for the last 15 min of the incubation.

### Viral RNA quantification

The DENV2 copy number was quantified in the culture supernatants using one-step quantitative real-time PCR. Known copies of the viral RNA were 10-fold serially diluted to generate a standard curve. The viral RNA was extracted using the QIAmp viral RNAmini kit (QIAGEN, Germany), and the qRT-PCR was performed using a SYBR Green Master Kit (Qiagen, Germany). Triplicate reactions were performed for each sample, and a no template control was included as a negative control. Absolute quantification was performed using an ABI7500 machine (Applied Biosystems, Foster City, CA). The results were analysed using Sequence Detection Software Version 1.3 (Applied Biosystems, Foster City, CA). The percentage of viral inhibition (%) was calculated as follows: 100 – (viral copy number of treated cells/viral copy number of untreated cells) × 100.

### Statistical analysis

All the assays were performed in triplicate, and the statistical analyses were performed using GraphPad Prism version 5.01 (GraphPad Software, San Diego, CA). *P* values <0.05 were considered significant. The error bars are expressed as ± SD.

## Results

### The inhibitory potential of the Ltc 1 peptide against the DENV2 protease NS2B-NS3pro

**Figure 1:**
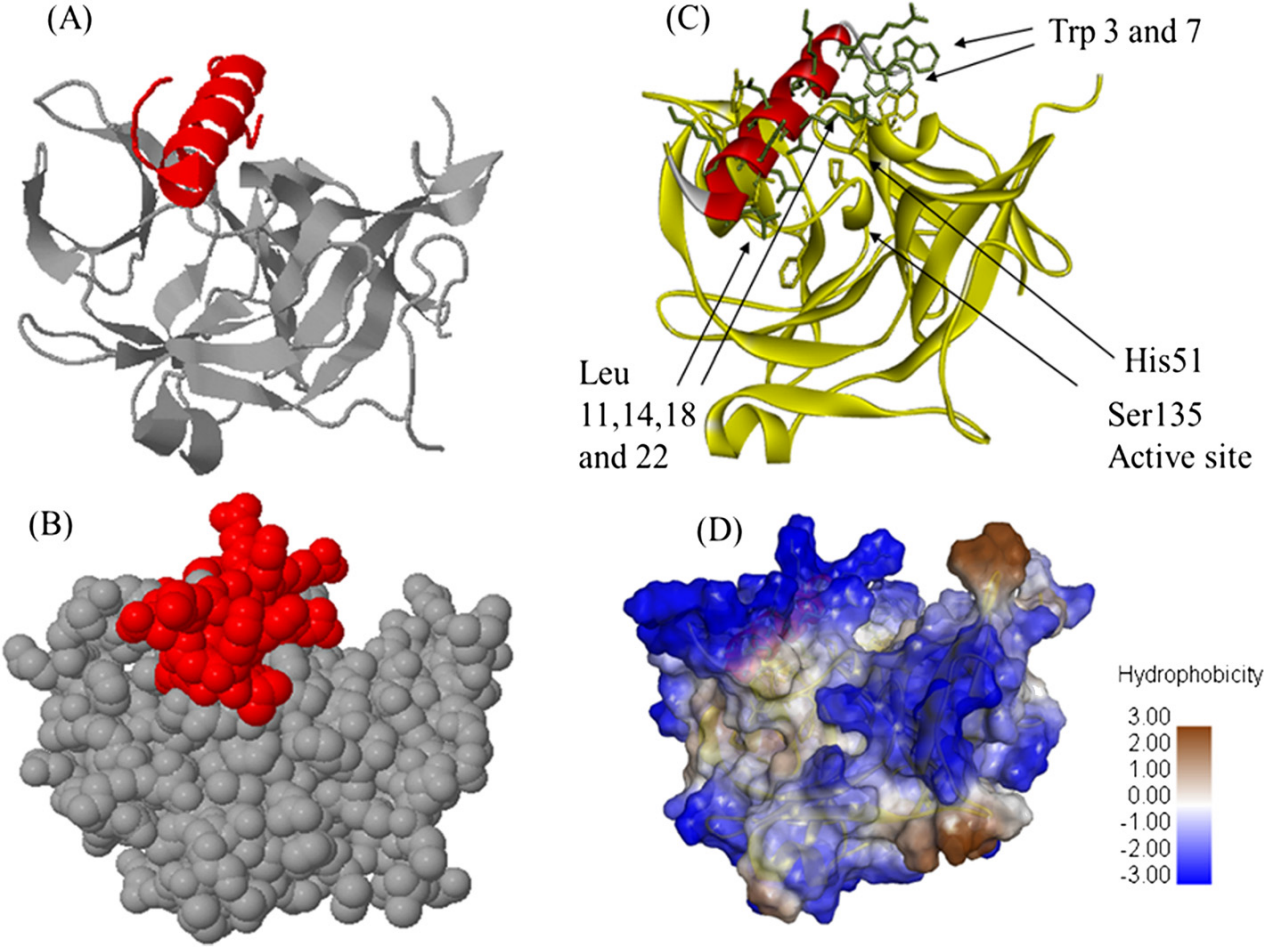
**Docking of Ltc 1 peptide with dengue NS2B-NS3pro. (A)** and **(B)** The results of the global rigid complementary docking performed using the FirDock online server showing the position of the Ltc 1 peptide (red) bound to the dengue NS2BNS3pro (grey) near the active site. **(C)** and **(D)** The results of Ltc 1 - dengue NS2B-NS3pro binding show the hydrophobic interaction of the four leucine and tryptophan residues of the Ltc 1 peptide (red) with the other hydrophobic residues of NS2B-NS3pro (yellow).

The results of the global rigid complementary docking showed that the Ltc 1 peptide bound the dengue NS2B-NS3pro near the active site (Figure 1A and 1B). The binding affinity depends on the hydrophobic interaction of four leucine residues and two tryptophan residues of the Ltc 1 peptide with the other hydrophobic residues of NS2B-NS3pro (Figure 1C and 1D). Therefore, a dengue NS2B-NS3pro assay was performed to confirm the docking findings that identified the possible interaction between the Ltc 1 peptide and the dengue NS2B-NS3 protease.

**Figure 2:**
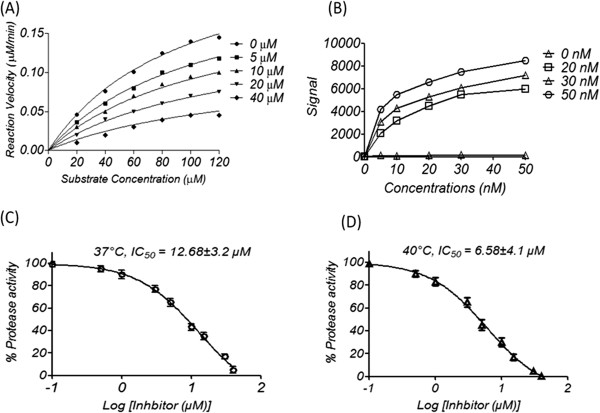
**Inhibitory effect of Ltc 1 peptides against dengue NS2B-NS3pro.** The recombinant dengue NS2B (G_4_-T-G_4_) NS3pro was produced as a recombinant protein in *E. coli*. **(A)** The kinetic assay plot for the inhibition of NS2BNS3pro from DENV2 by the Ltc 1 peptide. This assay was performed using increasing concentrations of inhibitor while all other conditions were kept constant. The data were analysed using the Michaelis-Menten model with a nonlinear regression curve fit in Graph Pad Prism (version 5.01) software. The concentrations of peptide were 0, 5, 10, 20, and 40 μM. **(B)** ELISA binding of Ltc 1 to dengue NS2B-NS3pro. Increasing concentrations of purified dengue NS2B-NS3pro (0, 20, 30 and 50 nM/well) were bound to black 96-well plate with transparent bottom. The Ltc 1 peptide labeled with FITC fluorescence dye (0, 0.1, 0.5, 1, 5, 10, 20, 30, 50 nM) were prepared in were bound to plates for 3 h on ice in dark place. the fluorescence signals of bound Ltc 1 were detected after washing steps using fluorescence spectrophotometer. **(C)** Determination of the IC_50_ value of the Ltc 1 peptide at normal physiologic human temperature (37°C). **(D)** Determination of the IC_50_ value of Ltc 1 peptide at the temperature of a human with a high fever (40°C).

Dengue NS2B-NS3pro was produced in *E. coli* as a recombinant protein, and its activity was evaluated using a fluorescent peptide substrate. After the optimisation steps, the results of this assay showed that the peptide exhibited significant dose-dependent inhibition of dengue NS2B-NS3pro (Figure 2A). The Ltc 1 peptide showed significant binding affinity to purifies dengue NS2B-NS3pro as evinced by ELISA binding assay (Figure 2B). The peptide showed higher inhibition of the dengue NS2B-NS3pro at a high fever-like human temperature (40°C) compared to normal physiologic human temperature (37°C). The inhibitory concentration of 50% of enzyme activity (IC_50_) was 6.58 ± 4.1 at 40°C compared to 12.68 ± 3.2 μM at 37°C (Figure 2C and 2D).

### The effect of the Ltc 1 peptide on cell proliferation and assessment of antiviral activity

**Figure 3:**
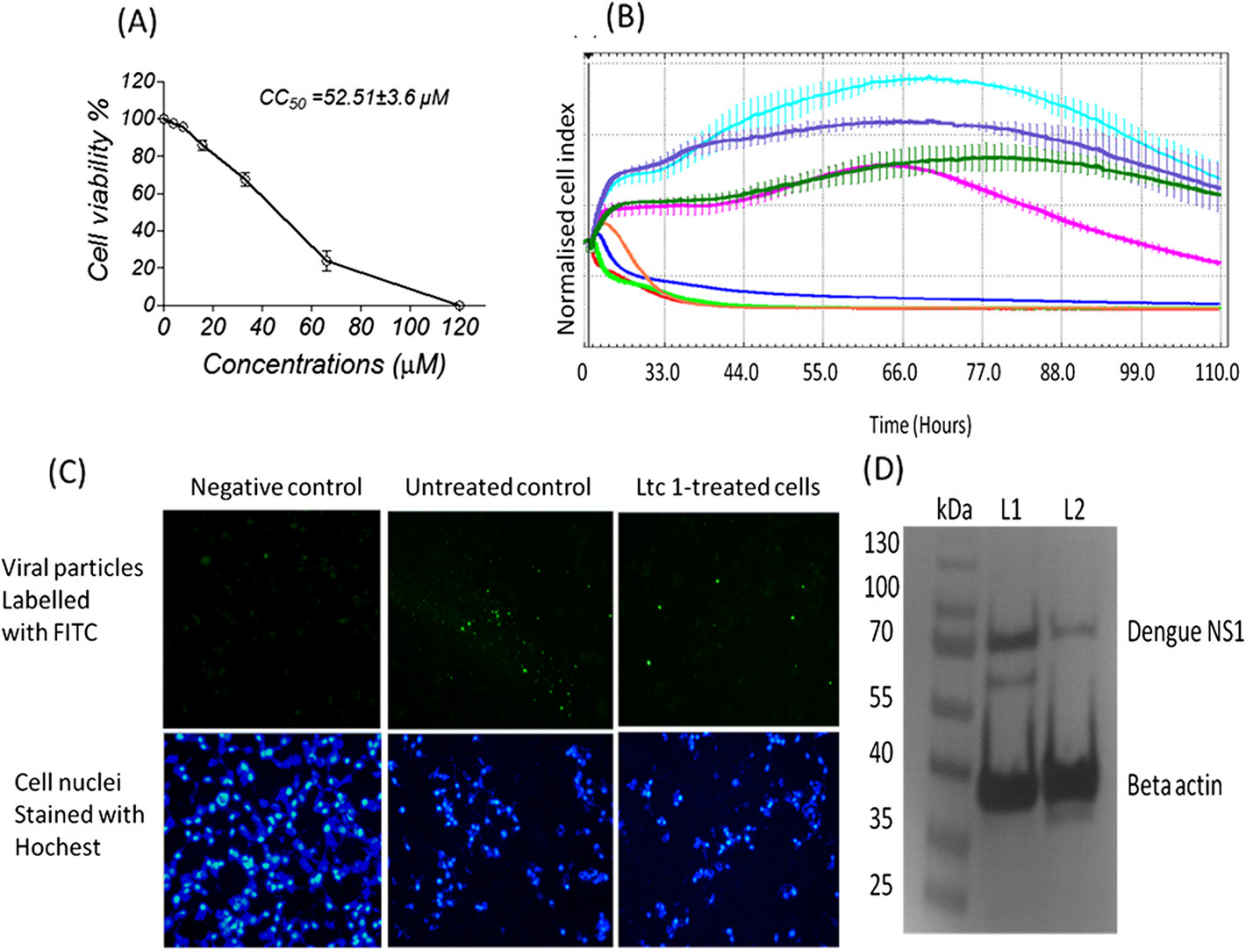
**Effect of the Ltc 1 peptide on cells proliferation and viral replication in HepG2 cells. (A)** The cytotoxic effect of the Ltc 1 peptide on cell viability was measured by non-radioactive cell proliferation assay. **(B)** The effect of the Ltc 1 peptide on cell proliferation was measured with the xCELLigence Real-Time Cellular Analysis (RTCA) system. Cell viability and growth were monitored continuously after applying increasing concentrations of the Ltc 1 peptide (0 (cyan), 12.5 (purple), 25 (dark green), 50 (magenta), 100 (orange), 150 (blue), 200 (green), and 250 μM (red)). **(C)** The effect of the Ltc 1 peptide on virus replication in infected cells. Viral particles were labelled with FITC fluorescence dye using indirect immunostaining, and the cell nuclei were stained with Hoechst. The figure shows a significant reduction of viral particles after peptide treatment. **(D)** Western blot analysis of the DENV2 NS1 protein expression level normalised to beta-actin as a reference cell protein (L1, untreated control; L2, DENV2-infected cells treated with Ltc 1 peptide).

The cytotoxic effect of Ltc 1 peptide on cell viability was measured using a non-radioactive cell proliferation assay. The CC_50_ value of the Ltc 1 peptide obtained via the optimisation steps was estimated to be approximately 52.51 ± 3.6 μM as shown in Figure 3A. The Ltc 1 peptide induces cellular changes that lead to cell apoptosis [21]. This activity may decrease the formation of plaques leading to a false interpretation of antiviral activity. To clarify this issue, we examined the effects of increasing concentrations of peptide on real time cell proliferation using the Real-Time Cellular Analysis (RTCA) system. The results showed that the effects of the peptide on cell proliferation were insignificant at 25 μM for 110 h because the cell index was similar to the untreated control cells. Cell proliferation was significantly decreased at 50 μM after 66 h of incubation of the HepG2 cells with the peptide (Figure 3B).Concentrations higher than 50 μM peptide were toxic to the cells at all time-points of the RTCA assay. Therefore, a concentration of 25 μM was identified as the maximal non-toxic dose (MNTD) of the Ltc 1 peptide used in the following experiment to evaluate the antiviral activity of the Ltc 1 peptide. The antiviral activity of the Ltc 1 peptide was initially evaluated by immunostaining and western blot targeting the DENV2 NS1 protein. The results showed a significant reduction of viral particles after treatment with the Ltc 1 peptide (Figure 3C). This result was further confirmed by western blot analysis that showed significant reduction in the expression of the viral NS1 protein after treatment of the infected cells with peptide. This result was normalised to beta-actin as an endogenous gene to eliminate loading errors (Figure 3D).

### Determination of antiviral inhibitory dose

**Figure 4:**
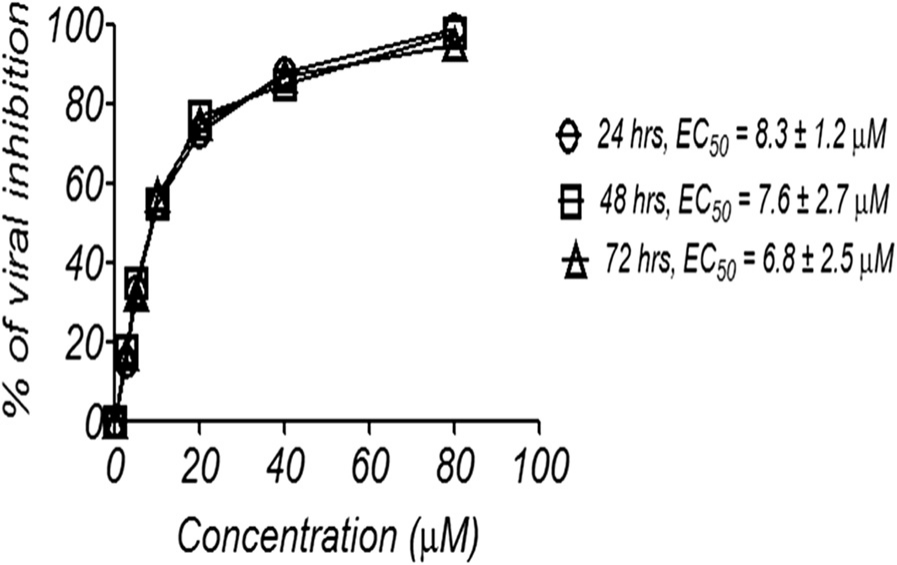
**Determination of viral inhibitory dose of the Ltc 1 peptide by RT-qPCR.** Serial concentrations of the Ltc 1 peptide (0, 2.5, 5, 10, 20, 40, and 80 μM) were incubated with HepG2 cells infected with DENV for 72 h. The viral RNA was quantified by one-step qRT-PCR. The results showed a dose-dependent reduction in viral copy number after treatment with the Ltc 1 peptide for 24, 48 and 72 h.

Quantitative real-time PCR was used to determine the viral copy numbers in the infected cells after treatment with the Ltc 1 peptide. The infected cells were treated with increasing concentrations of the Ltc 1 peptide for 24, 48 and 72 h. The Ltc 1 peptide showed dose-dependent inhibition of DENV2 replication in HepG2 cells. However, the results showed insignificant effects for the time points on peptide activity (Figure 4). The inhibitory effects of the Ltc 1 peptide were dependent on increasing concentrations of the peptide at the three time points. The Ltc 1 peptide inhibited DENV2 replication at EC_50_ values of 8.3 ± 1.2 μM for 24 h, 7.6 ± 2.7 μM for 48 h and 6.8 ± 2.5 μM for 72 h (Figure 4).

### The mode of inhibition

**Figure 5:**
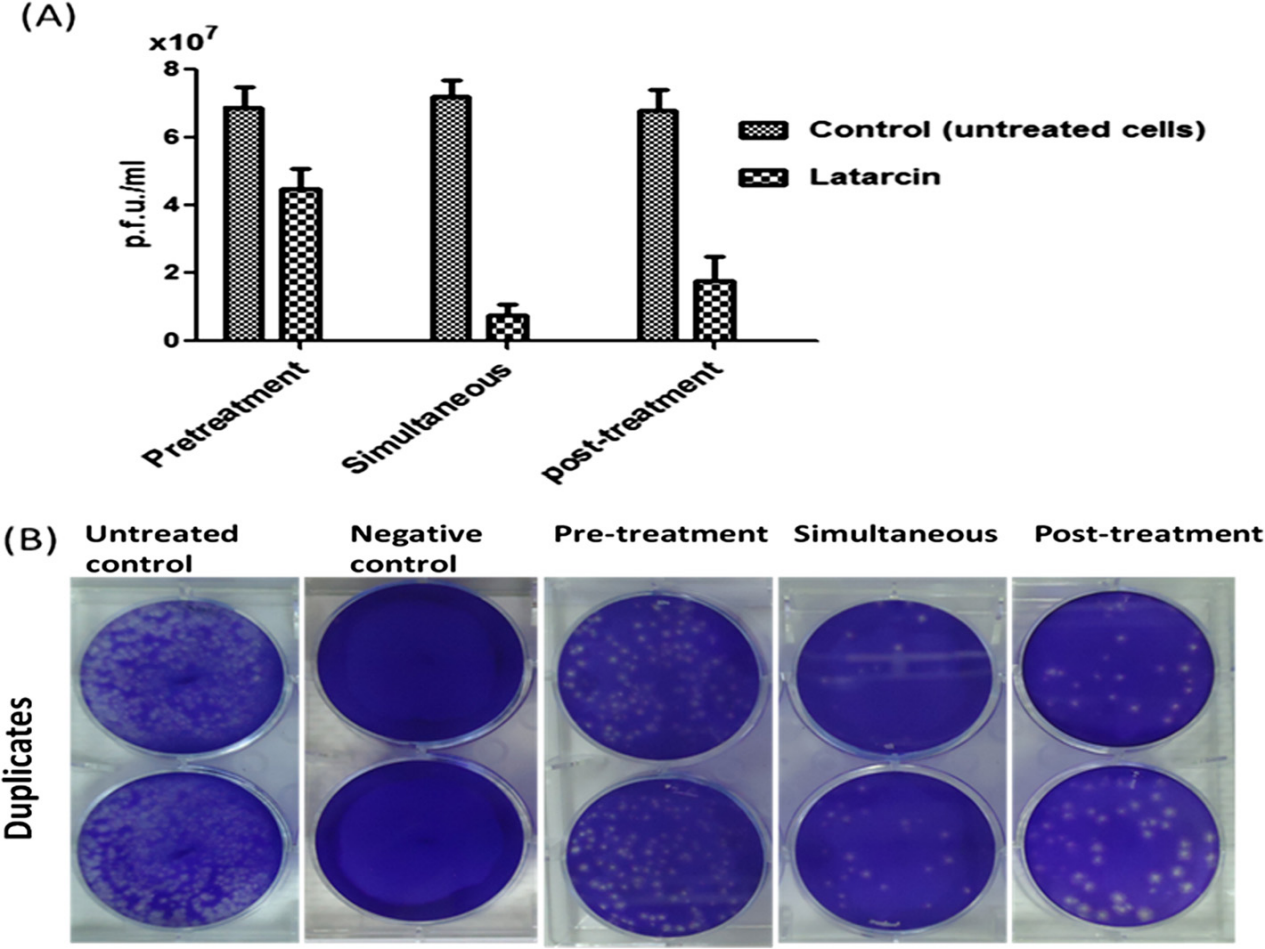
**Mode of action of the Ltc 1 peptide against DENV2 infection. (A)** The DENV2 viral load for the pre-, simultaneous and post-infection treatments with the latarcin peptide. The viral load (pfu/ml) was significantly reduced for the pre-treatment (4.5 ± 0.6 vs. 6.9 ± 0.5 control), simultaneous (0.7 ± 0.3 *vs.* 7.2 ± 0.5 control) and post-treatment (1.8 ± 0.7 *vs.* 6.8 ± 0.6 control) (two-way ANOVA with Bonferroni post-test). **(B)** The viral loads in the infected HepG2 cells of the pre-, simultaneous and post-infection treatments were quantified and calculated based on plaque formation in Vero cells after a five-day incubation.

The antiviral activity of the Ltc 1 peptide was further verified by plaque formation assay that showed different inhibitory effects of the peptide against virus entry and replication in infected cells. The Ltc 1 peptide showed significant inhibitory effects at a pre-treatment, simultaneous and post-treatment compared to the untreated cells. However, the antiviral activity for the simultaneous and post-treatment was significantly higher than the pre-treatment (Figure 4A). The viral load (pfu/ml) was significantly (*p* < 0.001) reduced at pre-treatment (4.5 ± 0.6) compared to the untreated cells (6.9 ± 0.5). In addition, a significant decrease (*p* < 0.0001) in viral load was observed for the simultaneous treatment (0.7 ± 0.3 *vs.* 7.2 ± 0.5 control) and post-treatment (1.8 ± 0.7 *vs.* 6.8 ± 0.6 control) as shown in Figure 5A and 5B.

## Discussion

We performed this study to identify and characterise the inhibitory potential of the latarcin peptide (Ltc 1) against dengue virus propagation in human cells. The results of the protein-protein docking study showed that the Ltc 1 peptide bound to the NS3 by hydrophobic residue interactions of the peptide, primarily Leu 11, 14, 18 and Trp 3 and 7 that interact with the surrounding hydrophobic residues of NS3 (Leu 28, Phe 30, Trp 50, Val 154 and Tyr 161). The binding of Ltc 1 to NS3 may effectively inhibit binding of the substrate to the active site or decrease the contribution of the NS2B co-factor active site formation. This observations were further considered by ELISA binding assay that showed significant binding affinity of Ltc 1 peptide to dengue NS2B-NS3pro.

The result of this study was further verified using a dengue NS2B-NS3pro assay that showed significant inhibition by the Ltc 1 peptide against dengue protease. Dengue NS2B-NS3pro cleaves the viral polyprotein at the positions between the capsid, NS2A-NS2B, NS2B-NS3, NS3-NS4A and NS4B-NS5, which lead to the release of mature individual viral structural (S) and non-structural (NS) proteins [6-9]. Therefore, inhibition of dengue NS2B-NS3pro may directly lead to inhibition of the post-translational processing of the viral polyprotein and subsequent virus replication [10,11].

In this study, the Ltc 1 peptide inhibited dengue NS2B-NS3pro in the low micromolar range (IC_50_ values of 12.68 μM at 37°C and 6.58 μM at 40°C). We hypothesise that the activity of the dengue protease decreased at the high fever temperature (40°C) because of the instability of the structural complex. Therefore, the Ltc 1 peptide showed higher inhibition, which is an approximately one fold reduction in the IC_50_ value compared to the inhibitory potential at 37°C. The activity of the NS2B-NS3pro primarily depends on the interaction between NS3 with the cofactor NS2B, which stabilises the enzyme structure and contributes to the formation of the active site [27,28].

Previous studies reported various inhibitors against dengue protease, including standard serine protease inhibitors [29], substrate based inhibitors [30], and non-substrate based inhibitors [31,32]. For example, aprotinin, a 58 amino acid protein, showed the highest inhibitory effect against the dengue protease at picomolar levels compared to the other standard serine protease inhibitors [33]. The peptidic α-keto amide [30] and cyclopeptide inhibitors [34] showed significant inhibition against dengue NS2B-NS3pro at micromolar dose ranges compared to the other peptidic inhibitors. In addition, the non-substrate based inhibitors, such as small molecule inhibitors, showed significant inhibitory activities at low micromolar concentrations against the flavivirus proteases [31,32]. Although several of these compounds are potent inhibitors of the dengue NS2b-NS3 protease, some showed poor stability in solution. Furthermore, several studies did not use cell-based assays to evaluate the toxicity and antiviral efficacy of the identified compounds [18]. The nature of the dengue protease, which possesses a flat and hydrophilic active site, decreases the possibility of identifying potent inhibitors to develop as antiviral therapeutics [18]. Based on the results of this study, we postulate that the hydrophobic residues of Ltc 1 are important for stabilising the binding to the hydrophilic active site of the dengue protease.

In this study, the inhibitory potential of the Ltc 1 peptide against the dengue protease was further verified using cell based assays. Previously, other characteristics of the latarcin family peptides, such as anti-neoplastic cells activities [21], were examined. The latarcin peptides can alter the lipid bilayers of the cell membrane, may induce the apoptosis of mammalian cells [21]. Because of this, the possible effect of the Ltc 1 peptide on cell proliferation was removed to avoid false interpretation of the antiviral activity. Subsequently, the antiviral activity of the Ltc 1 peptide was evaluated at the doses with minimal effects on cell proliferation as determined by MTT assay and Real-Time Cellular Analysis (RTCA). The results of the immunostaining and western blot analyses showed that the Ltc 1 peptide significantly reduced the viral particles and non-structural protein NS1 in DENV-infected cells. Furthermore, the results of the time-of-addition assay showed that the Ltc 1 peptide inhibited dengue virus replication at both the simultaneous and post-treatments compared to the pre-treatment. The mechanism of antimicrobial activity of the latarcin peptides depends on the helix-hinge-helix structure that is important for lysing bacterial cell membranes [35,36]. This finding emphasised that the direct incubation of DENV with the Ltc 1 peptide during the simultaneous treatment may led to lysis of the viral particles by the peptide. The results of the post-treatment and dose-response assays showed that the viral load was significantly deceased after treatment with the Ltc 1 peptide. Based on this finding, we hypothesise that the Ltc 1 peptide may interrupt the dengue life cycle in HepG2 cells during post-translational processing of the polyprotein by inhibiting the dengue serine protease. This inhibition may hinder flavivirus replication and virion assembly, as evidenced by the lack of infectious virion production in mutants carrying inactivating viral proteases [13]. However, other α-helical peptides, similar to Ltc 1, have significant inhibitory effects against HIV-1 because of an actual interference with the virus assembly stage of the viral life cycle [37]. Therefore, other mechanisms may be responsible for the observed effects, such as the interruption of virus assembly and release from the infected cells because of Ltc 1 interaction with other viral or host cell proteins; however, this warrants additional studies.

## Conclusions

The Ltc 1 peptide exhibited significant inhibition of the dengue protease and virus replication in HepG2 cells. Therefore, Ltc1 may act as a lead structure for developing therapies against DENV.

## Abbreviations

Ltc 1: Latarcin-1 peptide analogue; DENV2: Dengue virus serotype 2; NS2B: NS2B cofactor amino acids sequence 49–95 in DENV2 NS2B and 1394–1440 in DENV2 polyprotein; NS3pro: NS3 protease amino acids sequence 1–185 in NS3 protease and 1476–1660 in DENV2 polyprotein; NS2B-NS3pro: NS2B fused to NS3pro via 9 amino acids (G4-T-G4); AMC: Fluorogenic peptide substrate (Boc-Gly-Arg-Arg-AMC).

## Competing interests

The authors declare that they have no competing interests.

## Authors’ contributions

HAR designed and performed the experiments and drafted the manuscript. HB and MP participated in the experiments and data analysis. NSR and RY participated in the design and drafted the manuscript. All authors approved the final manuscript.
